# (3*S*)-1,2,3,4-Tetrahydro-β-carboline-3-carboxylic Acid from *Cichorium endivia*. L Induces Apoptosis of Human Colorectal Cancer HCT-8 Cells

**DOI:** 10.3390/molecules18010418

**Published:** 2012-12-28

**Authors:** Fu-Xin Wang, An-Jun Deng, Ming Li, Jin-Feng Wei, Hai-Lin Qin, Ai-Ping Wang

**Affiliations:** 1 New Drug Safety Evaluation Center, Institute of Materia Medica, Chinese Academy of Medical Sciences and Peking Union Medical College, No.1 Xiannongtan Street, Beijing 100050, China; E-Mails: wangfx@imm.ac.cn (F.-X.W.); weijinfeng@imm.ac.cn (J.-F.W.); 2 State Key Laboratory of Bioactive Substance and Function of Natural Medicine, Institute of Materia Medica, Chinese Academy of Medical Sciences and Peking Union Medical College, No.1 Xiannongtan Street, Beijing 100050, China; E-Mail: denganjun@imm.ac.cn; 3 Department of Microecology, Dalian Medical University, No.9 Western Section, Lvshun South Street, Lvshunkou District, 116044, Dalian, China; E-Mail: vivianmarat@163.com

**Keywords:** *Cichorium endivia*, (3*S*)-1,2,3,4-tetrahydro-β-carboline-3-carboxylic acid, NF-κB, apoptosis

## Abstract

*Cichorium endivia*. L, consumed either cooked or eaten raw in salads, is a popular kind of vegetable cultivated all around the World. Its components have been widely used in folk medicine in anti-inflammatory therapy. However, the anti-cancer activity of the components has never been reported. In this study, (3*S*)-1,2,3,4-tetrahydro-β-carboline-3-carboxylic acid (**1**), an amino acid isolated from *C. endivia*. L, was found for the first time to show cytotoxic activity in colorectal cancer cell line HCT-8. Compound **1** at concentrations of 0.5–4 μM induced apoptosis of HCT-8 cells in a dose-dependent manner. The compound **1**-induced apoptosis in HCT-8 cells was accompanied by the loss of mitochondrial membrane potential, the activation of caspase-3, caspase-8 and caspase-9, the up-regulation of Bax and the down-regulation of Bcl-2. In addition, compound **1** suppressed the activation of NF-κB, which acts as an inhibitor of apoptosis. Taken together, these results suggested that compound **1** could significantly induce apoptosis of HCT-8 cells through the suppression of NF-κB signaling pathway, and thus can be considered as a potential candidate for developing chemotherapeutic drugs against cancer.

## 1. Introduction

*Cichorium endivia*. L is a popular vegetable from the Compositae family and is widely cultivated all over the planet. It is very valuable nutritionally, with a high content of dietary fibre, potassium and vitamin C [1]. The natural components of this vegetable also have many biological activities and functions, such as anti-inflammation [2] and hepatoprotective effects [3]. This activity is mainly due to its high levels of antioxidant compounds [2]. In addition, discovery of active constituents such as sesquiterpenes and phenolic compounds from *C. endivia* has also been reported [4,5,6], suggesting a prospect of other interesting pharmacological applications. However, the activities of *C. endivia* components against carcinogenesis have never been reported.

Colorectal cancer (CRC) is the third most common cancer in both men and women, and it is the second leading cause of cancer-related mortality in the Western world [7]. In China, due to the changes of eating habits and aging problems, the disease incidence of CRC is increasing by 4.71% per year, which is far more than the international average (2%). Therefore, the research and development of potent preventive and anti-cancer agents against CRC are very urgent.

Many studies have shown a positive correlation between the consumption of vegetables and the decreased incidence of gastrointestinal tract tumors [8]. The components of certain plants were found to inhibit cell proliferation and induce apoptosis [9] by interacting with the apoptotic signaling pathways through activation of the caspase family of cysteine proteases [10], or the augmentation of Bax/Bcl protein levels [11]. Other mechanisms underlying the anticarcinogenic effect of plant components were also proposed, such as anti-inflammatory and antioxidant activities, the effect on NF-κB transcription factors [12], the inhibition of matrix metalloproteinase (MMP) [13], *etc.* Considering the antioxidant and many other biological activities of *Cichorium endivia*, we hypothesized that components of this plant may have anti-tumor activities against cancers such as CRC.

**Figure 1:**
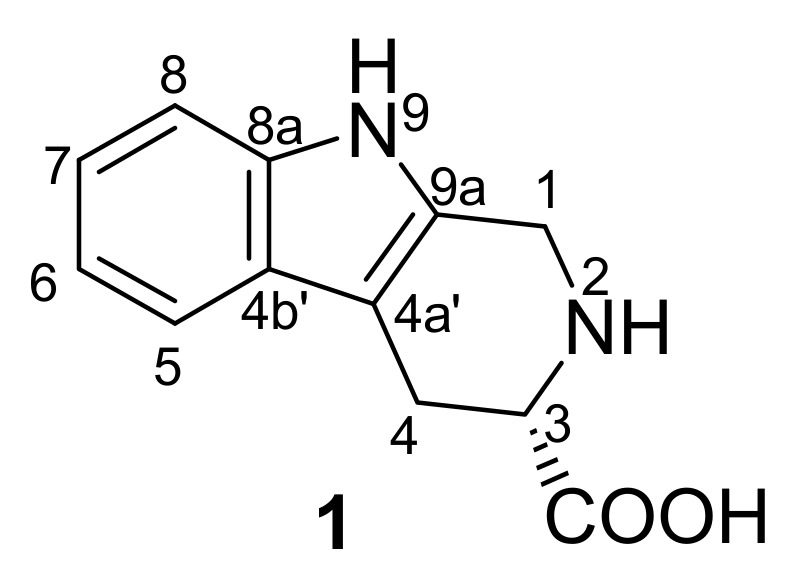
The structure of compound **1**.

Recently, we isolated a major constituent from *C. endivia*, (3*S*)-1,2,3,4-tetrahydro-*β*-carboline-3-carboxylic acid (**1**), an amino acid (C_12_H_12_N_2_O_2_, MW 216.09) (Figure 1), that showed high anti-proliferative effect on human CRC cell line HCT-8, and it strongly induced the apoptosis of HCT-8 cells in a dose-dependent manner. To our knowledge, this is the first study showing the anticancer activity of a natural component isolated from *C. endivia*. To further investigate the mechanism of the anti-proliferation and apoptosis-inducing activities of compound **1**, we analyzed the mitochondrial membrane potential of HCT-8 cells, the expression levels of Bax, Bcl-2, and caspase proteins under treatment of compound **1**. In addition, the activity of NF-κB, an inhibitor of apoptosis, was also detected. We expect that our results in this study will highlight the anti-cancer effects of *C. endivia* components, and provide a scientific basis for the development of natural plant components as chemotherapeutic drugs against CRC.

## 2. Results and Discussion

### 2.1. Structure Elucidation of Compound ***1***

The air-dried and pulverized *C. endivia* was extracted under reflux conditions with 95% EtOH and the concentrated solution was successively subjected to extraction with petroleum ether and EtOAc. The rest of the water soluble fraction was loaded on a column filled with Daion HP-20 and eluted with H_2_O, 60% EtOH, and 95% EtOH, respectively. The 60% EtOH eluates were evaporated under vacuum which yielded a black residue. The 60% EtOH fraction was re-dissolved in *n*-BuOH and washed with aq. 5% NaHCO_3_, then H_2_O, respectively. Evaporation of the *n*-BuOH solvent under reduced pressure gave 5.5 g of brown-green residue, which was applied to an ODS-A column and eluted with MeOH–H_2_O of decreasing polarity (40–100%) to yield compound **1** (0.108g) as a pale-yellow powder after removal of the solvent. The structure of **1** was elucidated as (3*S*)-1,2,3,4-tetrahydro-β-carboline-3-carboxylic acid on the basis of detailed spectroscopic analysis, including mainly 1D and 2D NMR and HRESI-MS, and comparison with the literature data [14]. IR (KBr) υ_max_ (cm^−1^): 3284, 3019, 2849, 1642, 1598, 1452, 1409, 1271, 1221, and 740; ESI-MS (positive mode) *m/z*: 217.1 [M+H]^+^ and 239.1 [M+Na]^+^; HRESI-MS (positive mode) *m/z*: 217.0967 [M+H]^+^ (calcd for C_12_H_12_N_2_O_2_, 217.0972). ^1^H- and ^13^C-NMR spectroscopic data are listed in Table 1.

### 2.2. Effect of Compound ***1*** on Viability of HCT-8 Cells

**Figure 2:**
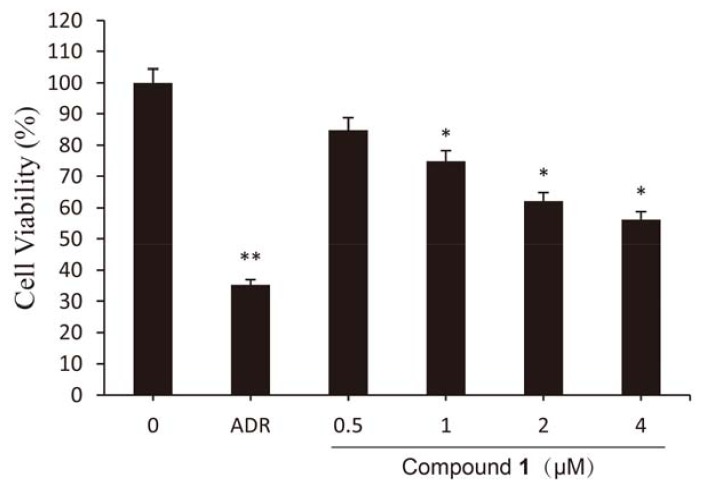
Viability of HCT-8 cells treated by compound **1** at different concentrations for 48 h. Results are expressed as mean ± SD (n = 3). * *p* < 0.05, ** *p* < 0.01, significantly different from the untreated group.

To determine the effect of compound **1** on growth of cancer cells, HCT-8 cells were treated with increasing concentrations of compound **1** for 48 h. As shown in Figure 2, compound **1** at a concentration of 4 μM decreased the viability of HCT-8 cells to 56.18%. From the results of a SRB assay, compound **1** was found to have a high anti-proliferative effect in HCT-8 cells compared with the control. However, the anti-cancer activity of compound **1** wasn’t stronger than that of adriamycin which used as a positive control (ADR, 2 μM). Beyond that, compound **1** induced cell death and the cytotoxic activity was dose-dependent (0.5–4 μM). The results mentioned above suggest that treatment with compound **1** resulted in strong inhibition of HCT-8 cell proliferation.

### 2.3. Compound ***1*** Induced the Apoptosis of HCT-8 Cells

**Figure 3:**
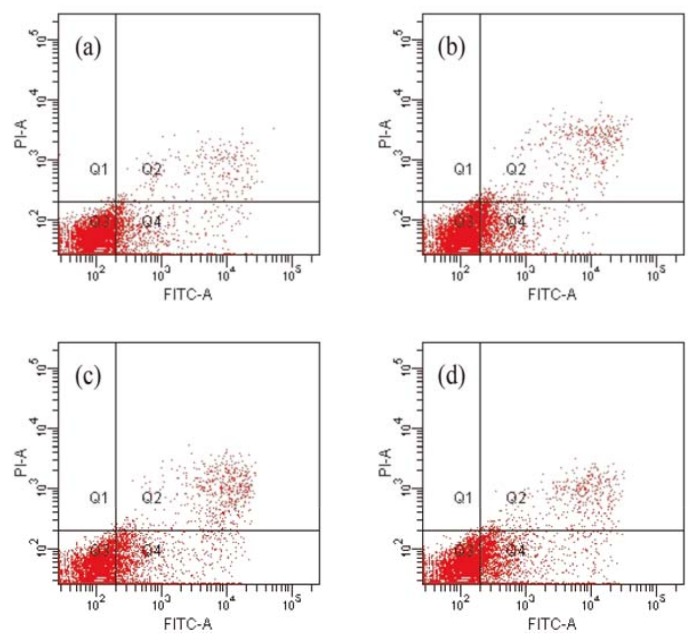
The fluorescence-activated cell sorter analysis for Annexin V/PI staining of HCT-8 cells treated with compound **1**. (**a**) Control, apoptotic rate 10.2%; (**b**) compound **1** 1 μM, apoptotic rate 14.4%; (**c**) compound **1** 2 μM, apoptotic rate 16.5%; (**d**) compound **1** 4 μM apoptotic rate 20.5%.

Apoptosis is an important cellular process that eliminates unwanted cells during normal development or damaged cells after removal of trophic factors or exposure to toxic chemicals [15]. Therapeutic strategies have targeted most of the key players in cellular apoptosis regulation [16]. Based on previous results of cell growth inhibition, we evaluated the rates of cell death to assess whether compound **1** is able to induce HCT-8 cell apoptosis. Cells were stained with annexin V/ propidium iodide (PI) and then measured by a flow cytometer. The intensity values for classification of the cells in positive and negative classes were determined from histogram analysis of signals from PI only and annexin V-FITC only. Results are shown in Figure 3. The lower left quadrants of the cytograms show the viable cells, which exclude PI and are negative for annexin V-FITC binding. The lower right quadrants represent the early apoptotic cells, annexin V-FITC positive and PI negative. The upper right quadrants contain late apoptotic and necrotic cells, positive for annexin V binding and for PI uptake. The upper left quadrants represent cells damaged during the procedure. As compared to the untreated group, the early apoptotic cell number significantly increased from 10.2% (control) to 20.5% following treatment with compound **1** (4 μM). The above changes could suggest that compound **1** induce HCT-8 cells apoptosis dose-dependently. 

### 2.4. Effects of Compound ***1*** on Expression of Bax and Bcl-2 Proteins in HCT-8 Cells

**Figure 4:**
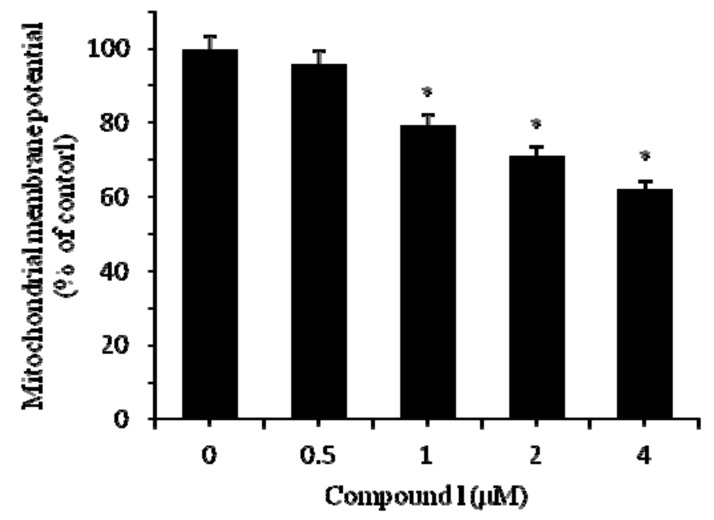
Mitochondrial membrane potential loss of HCT-8 cells treated by compound **1** at different concentrations for 24 h. Results are expressed as mean ± SD (n = 3). * *p* < 0.05, significantly different from the untreated group.

**Figure 5:**
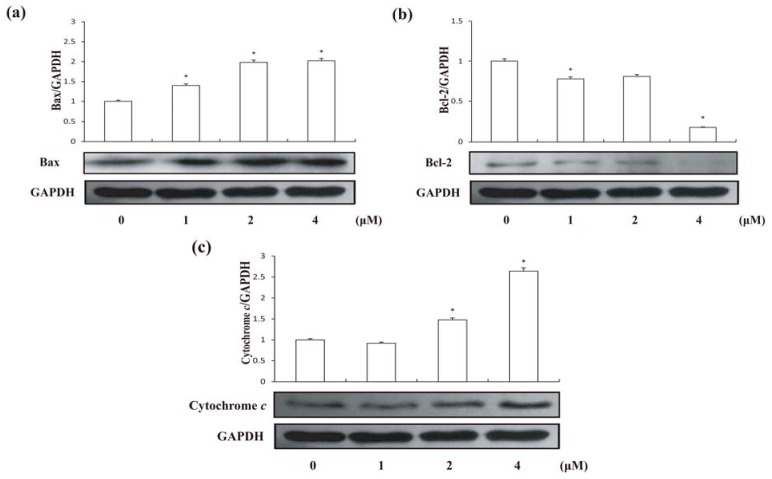
Expression levels of Bax and Bcl-2 in HCT-8 cells treated by compound **1** at different concentrations for 24 h; (**a**) Bax expression; (**b**) Bcl-2 expression; and (**c**) Cytosolic cytochrome *c*. Results are expressed as mean ± SD (n = 3). * *p* < 0.05, significantly different from the untreated group.

The Bcl-2 family of proteins contributes significantly to the mitochondrial pathway of apoptosis in response to diverse cytotoxic agents, and they are divided into two groups: suppressors of apoptosis (e.g., Bcl-2 and Bcl-xL) and activators of apoptosis (e.g., Bax and Bad). It is clear that Bcl-2 or Bax may control the mitochondrial permeability of the transition pores or other early mitochondrial perturbation [17]. Therefore, Bcl-2 or Bax may facilitate the passage of some important proteins, such as cytochrome *c* or other apoptosis inducing factors that trigger the activation of the caspase cascade resulting in apoptosis [18]. Disruption of the mitochondrial membrane potential is one of the earliest intracellular events that occur following the induction of apoptosis [19]. Under treatment of compound **1**, we found a significant decrease (62.09 ± 3% compared with untreated group) of mitochondrial membrane potential in HCT-8 cells after 24 h (Figure 4). Furthermore, we found that compound **1** significantly increased Bax in HCT-8 cells after 24vh treatment (Figure 5a). Moreover, treatments of compound **1** also lead to a decrease of Bcl-2 in HCT-8 cells (Figure 5b). In addition, cytochrome *c* in HCT-8 cells significantly increased after treated with 4 μM compound **1** for 24 h compared with control (Figure 5c). These suggested that compound **1** has damaged the mitochondrial membrane, which enhanced the expression of Bax and Bax enhancement in turn induced the loss of membrane potential, leading to cytochrome *c* release and the inhibition of Bcl-2.

### 2.5. Effects of Compound ***1*** on Caspase Activities

**Figure 6:**
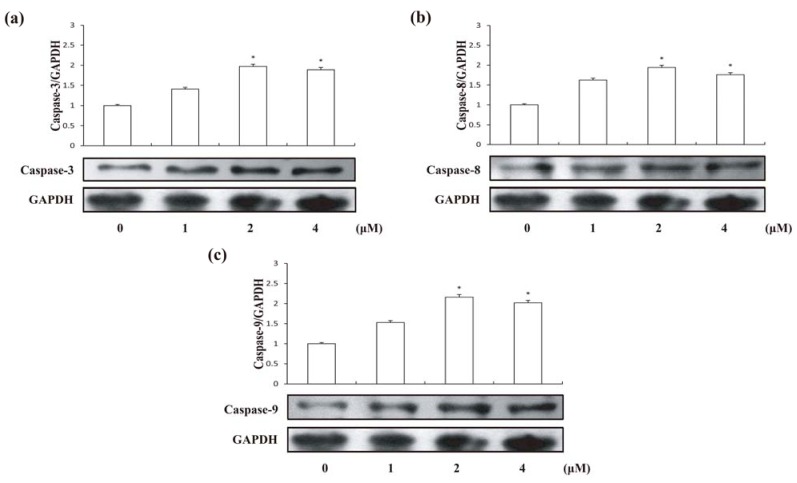
Expression levels of Caspases in HCT-8 cells treated by compound **1** at different concentrations for 24 h. (**a**) Caspase-3; (**b**) Caspase-8; (**c**) Caspase-9. Results are expressed as mean ± SD (n = 3). * *p* < 0.05, significantly different from the untreated group.

Apoptosis may occur via a death receptor-dependent extrinsic or a mitochondria-dependent intrinsic pathway [20]. The two apoptotic pathways both relate to the activation of the caspase proteases, which are aspartate-specific cysteine proteases. Caspase-8 and caspase-9 are activators/initiators of the TNF-α receptor and mitochondrial pathways, respectively [21]. Both of the main pathways involve caspase-3. However, during the process of caspase cascade activation, caspase-3 is the main executioner caspase inducing cell apoptosis [22]. Aiming to identify the mechanism of compound **1**-induced apoptosis, the cellular pathway of compound **1**-induced cell death was examined by assessing caspase activities. Following 24 h treatment of HCT-8 cells with different concentrations of compound **1**, enhancements of caspase-3, caspase-8 and caspase-9 activities were detected (Figure 6). 

### 2.6. Compound ***1*** suppressed the Activation of NF-κB

**Figure 7:**
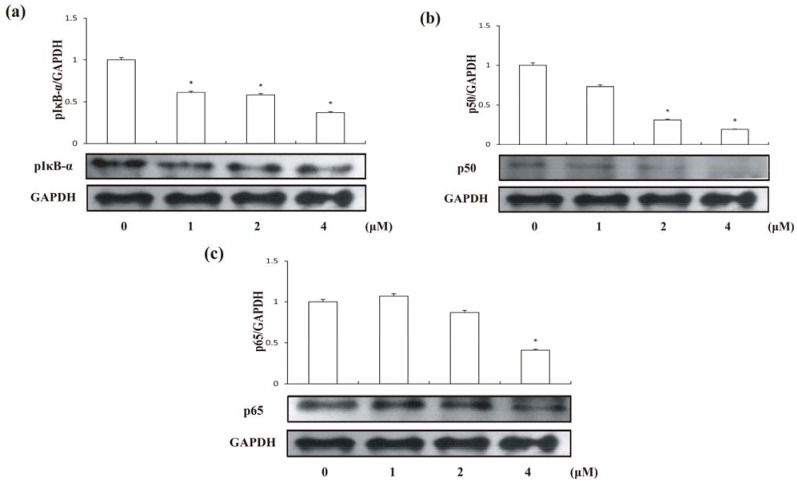
Expression levels of p50, p65 and phosphorylated IκB-α in HCT-8 cells treated by compound **1** at different concentrations for 24 h; (**a**) pIκB-α; (**b**) p50; (**c**) p65. Results are expressed as mean ± SD (n = 3). * *p* < 0.05, significantly different from the untreated group.

Recent evidence indicates that the activation of NF-κB plays an important role in coordinating apoptotic cell death [23]. Many anti-cancer drugs could activate intracellular signaling pathways involved in suppression of transcription factor NF-κB. The most common subunits of NF-κB complex expressed are p50, p65 and IκB-α. A family of inhibitory proteins, IκBs, binds to NF-κB (p50/p65 heterodimer) and masks its nuclear localization signal domain and therefore controls the translocation of NF-κB [24]. To examine whether inhibition of NF-κB is also associated with compound **1**-induced apoptotic cell death and further understand the mechanism, we assessed the effects of compound **1** on the expressions of p50, p65 and on phosphorylation of IκB-α by Western blotting. It is generally believed that the phosphorylation of IκB-α and the following release from p65/p50 heterodimer are the critical steps for activation of NF-κB [25,26,27], which the activated heterodimer then translocates into the nucleus where it could participate in the regulation of numerous genes transcriptions [28,29]. As showed in Figure 7, the phosphorylation of IκB-α was suppressed by the compound **1**, therefore p50/p65 heterodimer could not be activated. Further, compound **1** also decreased expression of p50 and p65 in cytosol dose-dependently. Based on the current results, we suggested compound **1** could suppress NF-κB signaling pathway by preventing p50/p65 heterodimer activation via inhibition of IκB-α phosphorylation in addition to repress the expression of its active components p65 and p50.

Studies have revealed that NF-κB activation plays an important role in coordinating the control of apoptotic cell death. It has been reported that several apoptosis-associated gens such as Bax, Bcl-2, and caspase-3 could be affected by NF-κB activation during cell apoptosis. Our results here are consistent with this conclusion.

## 3. Experimental 

### 3.1. Materials

(3*S*)-1,2,3,4-Tetrahydro-β-carboline-3-carboxylic acid is a pale-yellow powder, and was supplied by Prof. Hai-lin Qin of the Institute of Materia Medica, Chinese Academy of Medical Sciences and Peking Union Medical College. Compound **1** was dissolved in phosphate-buffered saline (PBS), and then diluted as needed in RPMI1640 medium (Hyclone, South Logan, UT, USA) immediately before use. The primary antibodies against IκB, p50, p65 were from Cell Signaling Technology (CST, Danvers, MA, USA), Bax, caspase-3, caspase-8, caspase-9 (all of the antibodies of caspases are active forms) GAPDH and Bcl-2 were from Santa Cruz Biotechnology (Santa Cruz, CA, USA). All other common chemicals were from Sigma Aldrich (St. Louis, MO, USA).

### 3.2. Cell Culture

Human colon cancer cell line HCT-8 cells were cultivated in RPMI1640 medium supplemented with 0.22% sodium bicarbonate, 10% inactivated fetal bovine serum (Hyclone), 100 U/mL penicillin and 100 μg/mL streptomycin and incubated at 37 °C in 5% CO_2_. 

### 3.3. Cell Viability Test (SRB assay)

The cell viability was measured by SRB (Sigma) assay. Briefly, the cells were seeded in 96-well plates (1 × 10^4^ cells/well) and routinely cultured for 24 h. Compound **1** was added in serial concentrations (from 0.5 μM to 4 μM); ADR (2 μM) was used as a positive control. While PBS was added alone as a negative control, and incubation was continued for an additional 48 h. SRB (1 mg/mL) was added to each well after the plates were fixed using TCA (0.4% m/v). After 20 min of incubation, each well was washed by acid (1% v/v) three times. Then Tris (100 mmol/L) was added into wells, respectively. The absorbance of each well was recorded on a microplate spectrophotomer at 515 nm.

### 3.4. Assessment of Apoptosis on HCT-8 Cells by Annexin V/PI Staining

HCT-8 cells (1 × 10^6^ cells/mL) were harvested, washed and double-stained by using an Annexin V-FITC apoptosis detection kit (Beyotime, Jiangsu, China). Cells which lost membrane integrity will show red staining PI throughout the nucleus and therefore will be easily distinguished between the early and the late apoptotic cells or necrotic cells. Samples were incubated at room temperature for 20 min in dark with annexin V and PI. Then samples were quantitatively analyzed by FAC Scan flow cytometer (Becton Dickinson, San Jose, CA, USA).

### 3.5. Analysis of Mitochondrial Membrane Potential

In order to testify the changes in mitochondrial membrane potential induced by compound **1** in HCT-8 cells, a fluorescent micro-plate was used to test the fluorescence intensity of Rh123, uptake of which by mitochondria is proportional to the mitochondrial membrane potential. Treatments with different dose of compound **1** for 24 h cause lowering of mitochondrial membrane potential.

### 3.6. Western Blot Analysis 

The cells were disrupted in lysis buffer (150 mM NaCl, 1.0% NP-40, 0.5% NaVO_4_, 0.1% SDS, 50 mM Tris, pH 7.5) containing 1 mM PMSF at 4 °C. After centrifugation for 30 min at 10,000 × *g* (4 °C), the protein concentration in the supernatant was determined using a BCA protein assay kit (Beyotime, Jiangsu, China). Samples containing equal amounts of protein were loaded onto 10% sodium dodecyl sulfate-polyacrylamide gel and transferred to polyvinylidene difluoride membranes. Membranes were blocked with 5% non-fat milk in 50 mM Tris-buffered saline containing 0.1% Tween 20 (TBS-T) for 1 h at room temperature and were then incubated overnight with antibodies at 4 °C. After washing in TBS-T three times, the membranes were incubated with secondary antibody at 37 °C for 1 h. The membranes were washed another three times with TBS-T and then visualized with ECL.

### 3.7. Statistical Analysis

The data of biochemical estimations were reported as means ± S.E, where n = 3. A one-way analysis of variance (ANOVA) followed by Student’s *t*-test were performed for determining whether individual doses were significantly relative to controls. A *p*-value less than 0.05 were considered statistically significant.

## 4. Conclusions 

In conclusion, the *C. endivia* component compound **1** could significantly inhibit the proliferation and induce apoptosis in HCT-8 cells. The compound **1**-induced apoptosis was associated with the suppression of NF-κB signaling pathway. This plant component can therefore be considered as a potential candidate for developing chemotherapeutic drugs against cancer, and its anticancer mechanism should be further explored.

## Figures and Tables

**Table 1:** ^1^H and ^13^C-NMR spectroscopic data of compound **1**.

No.	δ_H_ *^ab^*	δ_C_ *^ac^*	δ_H_ *^de^*
1a	4.38 d (15.6)	40.5 t	4.15 d (15.3)
1b	4.54 d (15.6)		4.23 d (15.9)
3	4.27 dd (10.4, 5.6)	55.0 d	3.60 m
4a	3.13 dd (10.8, 16.4)	21.7 t	2.81 dd-like
4b	3.38 dd (5.6, 16.4)		3.13 br d-like
4a′		104.9 s	
4b′		125.6 *^f^* s	
5	7.53 d (8.0)	118.2 d	7.32 d (7.5)
6	7.13 t (7.6)	120.0 d	7.06 t (7.5)
7	7.22 t (8.0)	122.9 d	6.97 t (7.8)
8	7.43 d (8.0)	111.9 d	7.43 d (7.5)
8a		136.7 s	
9a		125.4 *^f^*s	
COOH		171.3 s	10.91 s
9-NH			

^*a*^ D_2_O + drops of F3CCOOD; *^b^* 400 MHz. *^c^* 100 MHz. *^d^* in DMSO-d_6_; *^e^* 300 MHz. *^f^* Assignments may be interchanged.
